# Genetically Predicted Circulating Copper and Risk of Chronic Kidney Disease: A Mendelian Randomization Study

**DOI:** 10.3390/nu14030509

**Published:** 2022-01-25

**Authors:** Shafqat Ahmad, Johan Ärnlöv, Susanna C. Larsson

**Affiliations:** 1 Molecular Epidemiology and Science for Life Laboratory, Department of Medical Sciences, Uppsala University, EpiHubben, MTC-huset, 751 85 Uppsala, Sweden; 2 Preventive Medicine Division, Harvard Medical School, Brigham and Women’s Hospital, Boston, MA 02115, USA; 3 Division of Family Medicine and Primary Care, Department of Neurobiology, Care Sciences and Society (NVS), Karolinska Institutet, 141 52 Stockholm, Sweden; johan.arnlov@ki.se; 4 School of Health and Social Studies, Dalarna University, 791 31 Falun, Sweden; 5 Unit of Cardiovascular and Nutritional Epidemiology, Institute of Environmental Medicine, Karolinska Institutet, 171 77 Stockholm, Sweden; susanna.larsson@ki.se; 6 Unit of Medical Epidemiology, Department of Surgical Sciences, Uppsala University, 751 85 Uppsala, Sweden

**Keywords:** circulating nutrients, kidney related disease, estimated glomerular filtration rate, Mendelian randomization

## Abstract

Elevated circulating copper levels have been associated with chronic kidney disease (CKD), kidney damage, and decline in kidney function. Using a two sample Mendelian randomization approach where copper-associated genetic variants were used as instrumental variables, genetically predicted higher circulating copper levels were associated with higher CKD prevalence (odds ratio 1.17; 95% confidence interval 1.04, 1.32; *p*-value = 0.009). There was suggestive evidence that genetically predicted higher copper was associated with a lower estimated glomerular filtration rate and a more rapid kidney damage decline. In conclusion, we observed that elevated circulating copper levels may be a causal risk factor for CKD.

## 1. Introduction

Copper is an essential trace element obtained mainly from foods, such as shellfish, organ meats, whole grain products, seeds, and nuts, but recent studies suggest that circulating copper concentrations are also to some extent genetically determined [1]. Alteration of circulating trace elements in many body organs including the brain, heart, kidney, and liver have been involved in trauma related deaths [2,3]. Excessive dietary copper intake can lead to deposition of copper in the kidney and cause nephrotoxicity characterized by proximal tube necrosis that results through oxidative stress, cellular injury, and leads to a decline in kidney function [4]. However, the interplay between copper and kidney disease is bi-directional as imbalances in the homeostasis of circulating copper levels may also occur as a result of impaired renal excretion and changes in protein metabolism in patients with chronic kidney disease (CKD) [5]. In fact, the regulation of copper levels in CKD patients is important to prevent complications. In previous observational studies, elevated circulating levels of copper have been associated with CKD [4,6]. Since traditional observational epidemiological studies are prone to confounding and reverse causality, it remains to be firmly established whether higher blood levels of copper cause kidney damage, a more rapid decline in kidney function and an increased risk of CKD.

One of the approaches to infer the causal links between lifestyle factors and disease outcomes is Mendelian randomization. Mendelian randomization (MR) is a framework to test the causal connections between modifiable exposures in relation to different phenotypes, where exposure-associated genetic variants are used as instrumental variables. Considering the random assortment of genetic variants during conception, genetic variants are not generally associated with potential confounders in the exposure-outcome association [7]. There are no previous studies that have examined the causal relationship between circulating copper levels in association with CKD.

We hypothesized that elevated circulating copper levels increase the risk of CKD. To test this hypothesis, we conducted a primary analysis of the association between genetically predicted circulating copper levels and CKD. In secondary analyses, we investigated whether genetically predicted high circulating copper levels associate with lower estimated glomerular filtration rate (eGFR), increased kidney rapid decline, and kidney damage.

## 2. Materials and Methods

### 2.1. Study Design

The current study is based on the MR design. The MR approach is based upon three assumptions. First, the genetic instrumental variable should be associated with the exposure. Second, the genetic instrumental variable should be associated with the potential confounders. Third, the genetic instrumental variable should effect the outcome variable through the exposure. The hypothesis was tested using copper-associated genetic variants as instrumental variables through a two sample MR approach [1]. In addition, MR studies are not vulnerable to reverse causation because genetic variants cannot be changed by disease status.

### 2.2. Genetic Instruments

Genetic association estimates for circulating copper were extracted from a genome-wide association meta-analysis of three population-based cohorts comprising 6937 individuals of European-descent [1]. In this meta-analysis study, circulating copper levels were measured in both plasma and serum. That study identified two genetic variants (rs17564336 in *SELENBP1* and rs34951015 in *CP*) at genome-wide significance in positive association with circulating copper levels. Both of those variants were used as genetic instruments in this MR study.

### 2.3. Outcomes

To access the potential causal association between circulating copper levels and CKD risk, genetic association estimates for CKD were obtained from a meta-analysis of genome-wide association studies (GWAS) with a total of 12,385 CKD cases and 104,780 non-cases of European descent [8]. CKD in that GWAS was defined as eGFR based on serum creatinine <60 mL min^−1^ per 1.73 m^2^. Genetic association estimates for eGFR, as a continuous variable, were obtained from a GWAS of 312,468 individuals of multiple ancestry (discovery dataset) [9] as well as the CKDGEN consortium and the UK Biobank cohort (*n* = 1,004,040) (replication dataset) [10].

To assess the potential causal link between circulating copper levels and the risk of creatinine based rapid decline of glomerular filtration rate (eGFRcrea), genetic association estimates for eGFRcrea were obtained from Gorski et al. [11]. The assessment of eGFRcrea was based on the sample from the CKDGEN consortium and UK Biobank cohort. Rapid eGFRcrea cases (also called CKDi25 including N_cases_ = 19,901 and N_controls_= 175,244) were defined as individuals who started with a baseline eGFR ≥ 60 mL/min per 1.73 m^2^ and decreased by >25% from this baseline to an eGFR < 60 mL/min per 1.73 m^2^, while the “CKDi25” controls were the individuals who started with a baseline eGFR > 60 mL/min per 1.73 m^2^ and did not meet these kidney function decline parameters (had not experienced 25% decline in eGFR) [11]. The mean eGFR decline follow-up time varies (from 1 to 15 years) in the participating study cohorts. Serum creatinine was measured using the enzymatic method as well as Jaffe reaction based method across the participating study cohorts. The estimation of eGFR was performed using the Chronic Kidney Disease Epidemiology Collaboration (CKD-EPI) equation [11].

To examine the potential causal link between circulating copper levels and kidney damage, genetic association estimates for markers of kidney damage were obtained from a GWAS meta-analysis by Teumer et al. [12]. Kidney damage was reflected by urinary albumin-to-creatinine ratio (*n* = 48,589) and normo-albuminuria vs. micro-/macro-albuminuria (*n* = 48,255) [12].

All the included study samples were based on participants of European ancestry, of age > 18 years, and contain both men and women. All the included studies were conducted following the standards of the Declaration of Helsinki.

### 2.4. Statistical Analysis

We used the inverse-variance weighted (IVW) method to estimate the causal estimates for the associations of genetically predicted copper with the kidney outcomes. We were unable to rule out potential pleiotropic effects by applying other MR methods, such as the weighted median and MR-Egger regression methods, as the minimum required number of genetic variants for these MR methods should be at least three, and we only had two copper-associated genetic variants. Analyses were conducted using MR base (https://www.mrbase.org/) (accession date: 26 December 2021) [13] and R-studio. *p*-value < 0.05 was considered to be statistically significant.

## 3. Results

**Figure 1:**
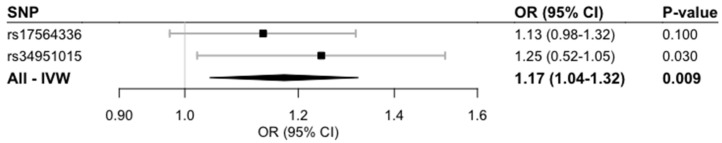
Associations of genetically predicted circulating copper with chronic kidney disease. CI, confidence interval; IVW, inverse variance weighted; OR, odds ratio; SNP, single-nucleotide polymorphism.

Genetically predicted higher circulating copper levels were significantly associated with an increased CKD prevalence (odds ratio 1.17; 95% confidence interval [CI] 1.04, 1.32; *p*-value = 0.009) (Figure 1).

Genetically predicted higher circulating copper levels were significantly associated with lower eGFR in the discovery study (beta −0.88 mL min^−1^ per 1.73 m^2^; 95% CI −1.72, −0.03; *p*-value = 0.043) [9] but were not significantly associated in the replication study (beta −0.0001 mL min^−1^ per 1.73 m^2^; 95% CI −0.0023, 0.0023; *p*-value = 0.91).

Our MR analysis provided evidence that genetically predicted higher circulating copper levels were marginally associated with increased decline of the glomerular function rate (odds ratio 1.10; 95% CI 0.99,1.23; *p*-value = 0.076).

Genetically predicted copper levels were not significantly associated with kidney damage when modelling urinary albumin-to-creatinine ratio as a continuous variable (beta 0.03; 95% CI −0.03, 0.06; *p*-value = 0.36), nor when comparing normo-albuminuria vs. micro-/macro-albuminuria (beta 0.05; 95% CI −0.34, 0.44; *p*-value = 0.80) [12].

## 4. Discussion

In the current study, we investigated the causal relationship between genetically predicted circulating copper and the risk of CKD, eGFR, kidney decline function, and urinary albumin-to-creatinine ratio and microalbuminuria. To the best our knowledge, this is the first study using the MR method to explore the causal connections between circulating copper intake and the CKD related outcomes. Our two-sample MR analysis confirms the observational association between high circulating copper levels and increased risk of CKD [4,6]. High circulating copper levels have also been associated with reduced eGFR, rapid kidney function decline, and higher levels of urinary albumin-to-creatinine ratio and microalbuminuria [14,15,16,17]. However, through MR analysis, we observed a nominally significant association of genetically predicted copper levels with reduced eGFR and rapid kidney function decline, but no association was observed with albuminuria.

Our important finding is that circulating copper was causally associated with higher risk of CKD prevalence and reduced eGFR. Copper is the important transition metal in humans and is a cofactor of several enzymes that are involved in many physiological pathways. Circulating copper is somewhat genetically controlled [1], but humans are also exposed to copper through dietary intake and it reaches the kidney through blood circulation [18]. In the kidneys, copper catalyzes the production of highly reactive hydroxyl radical species, so this oxidative stress can cause proximal tube necrosis [5,18]. Previously, through observational epidemiological studies, it has been reported that higher copper intake has been associated with abnormal eGFR [16] and end stage CKD [19]. Our study also proposed that circulating copper is causally associated with higher CKD prevalence as well as reduced eGFR. In the replication study (Stanzick et al.) [10], the eGFR-GWAS analysis was additionally adjusted for confounding factors, including interaction terms for sex*age, age^2^*sex. This might explain why the single nucleotide polymorphism-eGFR estimates in the analysis based on the replication study were very small in magnitude and not comparable with the estimates obtained based upon the GWAS by Morris et al. [9], where the regression estimates were not adjusted for any interaction terms.

Strengths of the current MR study are that circulating copper levels were measured through plasma and serum and that the genetic association estimates for copper and CKD were measured in independent samples, thereby avoiding bias in the direction of the observational association. The limitation of the current study is that only two genetic variants were used as instrumental variables. It was thus not possible to rule out potential pleiotropic effects using other MR methods that are more robust for such effects. Circulating copper levels were estimated through whole blood, serum, and erythrocytes with different transformation of copper values including varying model adjustment in the different cohorts of copper genome-wide association studies. Our study might be underpowered to detect weak causal connections between copper and renal biomarkers. Furthermore, we tested many outcome traits in MR analysis. However, even if we apply multiple correction, our results for genetically predicted circulating copper levels in association with CKD risk are statistically significant. In the replication sample of CKDGEN and UK Biobank, we observed directionally similar circulating copper-CKD estimates. We only use GWAS summary data and we are not able to define CKD using both GFR and albuminuria. Finally, our studies sample was based upon individuals of European ancestry, which limited generalizability to other ancestries.

## 5. Conclusions

In conclusion, findings of this MR study suggest that genetically predicted elevated circulating copper levels may be a causal risk factor for CKD and possibly reduced eGFR and rapid kidney decline function. Additional studies are needed to assess the clarification of potential underlying mechanisms and clinical relevance of these findings.

## Data Availability

As the current study used published GWAS summary data, so, the relevant data is publically available.
